# Assessment of Insomnia Among Emergency Department Physicians in the Western Region of Saudi Arabia, 2023

**DOI:** 10.7759/cureus.40721

**Published:** 2023-06-21

**Authors:** Ahmed Othman, Abdulhadi Tashkandi, Hattan Esilan, Ghadeer M Alfeheadi, Sadeem A Alqusair

**Affiliations:** 1 Emergency, Prince Mohammed Bin Abdulaziz Hospital, Madinah, SAU; 2 Emergency Medicine, Prince Mohammed Bin Abdulaziz National Guard Hospital, Medinah- AlMunawarah, SAU; 3 Emergency Medicine, Prince Mohmmed Bin Abdulaziz Hospital, Madinah, SAU; 4 Emergency Medicine, Prince Mohammad Bin Abdulaziz Hospital, Madinah, SAU; 5 Emergency Medicine, King Abdulaziz Medical City, Jeddah, SAU

**Keywords:** sleep, healthcare workers, insomnia, physicians, emergency medicine

## Abstract

Background

Sleep is essential for physical and mental health, and insomnia is the most common sleep disorder. Insomnia is associated with multiple health issues that may affect physicians' health and their decision-making, and subsequently affect patients in the emergency department (ED). This study aimed to assess insomnia and its associated factors among ED physicians in the western region of Saudi Arabia.

Methods

A cross-sectional study was conducted using a validated online questionnaire to collect data from the target population. The questionnaire included demographic characteristics, the nature of the job, shift characteristics, and the Insomnia Severity Index tool. Data were analyzed using the statistical package for the social sciences (SPSS, version 29.0), and the Chi-square test was used for bivariate analysis.

Results

The study involved 106 ED physicians, primarily from Saudi Arabia, who worked in government hospitals and were mostly aged from 20 to 29 years old. Of the participants, 35.8% had moderate to severe clinical insomnia, with younger physicians experiencing more severe insomnia than their older colleagues. This highlights the potential role of age in the development of insomnia. No significant associations were found between insomnia severity and other demographic or work-related factors.

Conclusion

This study found a high prevalence of insomnia among ED physicians in Saudi Arabia, emphasizing the need for further research and interventions to address this issue. Improving the sleep quality of ED physicians is crucial for their health and well-being, as well as for the safety and quality of care provided to their patients.

## Introduction

Sleep is a fundamental human need and a crucial component of both physical and mental health. It is vital for restoring energy consumed during the day and plays a key role in maintaining metabolic and endocrine functions, as well as improving cognitive functions like memory [1-4]. The term “insomnia” has multiple meanings in the literature and media. In survey studies, it is usually identified as a “Yes” response to questions like “Do you experience difficulty sleeping?” or “Do you have difficulty falling or staying asleep?” However, in medical literature, insomnia is often defined as the presence of polysomnographic evidence of sleep disturbance, which can be described by a long latency of sleep, recurrent nighttime awakening, or prolonged wakeful periods during sleep [5,6].

Insomnia, the most common sleep disorder, places a significant burden on vulnerable patient populations and the United States (US) healthcare system [7]. The total direct and indirect costs of insomnia in the United States exceed $100 billion annually [4]. The yearly loss of quality-adjusted life years from insomnia appears to be greater than the loss due to other medical and psychiatric disorders such as arthritis, depression, and hypertension [8]. Between 1993 and 2015, the number of insomnia diagnoses increased 11-fold [9]. Insomnia is also linked to higher medical care costs, particularly in patients with coexisting medical or psychiatric conditions [10,11]. The third edition of the International Classification of Sleep Disorders (ICSD-3), the most commonly used classification system for sleep disorders, revised the definition of insomnia to include short-term, chronic, and other subcategories [12]. The previous subclassification of chronic insomnia as a primary or comorbid disorder was removed because it did not improve diagnostic accuracy or distinguish between treatment alternatives. The underlying reason for the removal is that labeling insomnia as comorbidity can be misleading as it is a secondary problem that can be resolved with proper treatment of coexisting comorbidities. On the contrary, maladaptive cognition and behavior that perpetuate insomnia must be addressed independently of coexisting medical or psychiatric conditions [12].

Insomnia is a debilitating condition with numerous negative consequences on patients and the healthcare system. Shift workers, including eight and 12 hours shifts, are more prone to insomnia, lack of sleep, and circadian disorders that can affect physical and mental health compared to daytime workers [13]. A study in the US showed that 10% of shift workers complain of sleep disturbance [14]. In Korea, a shocking 55% of shift workers reported experiencing insomnia [15]. A different study in the US estimated the prevalence of insomnia and other sleep-related issues among 62 nurses and found that most nurses (92%) have one or more sleep-related problems, and insomnia was the most commonly reported concern [16]. Another study attempted to assess sleep disorders among healthcare workers in Saudi Arabia and found that among the 163 participants, 62 (38%) had insomnia as they reported inadequate sleep [17].

Emergency department (ED) physicians are the frontline workers in hospitals, encountering critical cases and dealing with a huge number of ED visits. They are assigned as team leaders and considered the most responsible person in the ED. According to the literature, shift workers are vulnerable to several sleep disorders, one of which is insomnia. Furthermore, insomnia is associated with multiple health issues that may affect physical health and their decision-making, and subsequently affect patients in the ED. Therefore, this study aims to assess insomnia and its associated factors among ED physicians in Saudi Arabia in 2023.

## Materials and methods

This is an analytical cross-sectional study that was carried out in the western region of Saudi Arabia. This study was conducted using an online questionnaire to collect data. ED physicians who are enrolled in the residency training program and currently on shift duties in the Western Region of the Kingdom of Saudi Arabia were invited to participate through a convenient sampling technique. The sample size was estimated to be 111 using a web-based sample size calculator (Raosoft.com), with a 5% margin of error, 95% confidence level, and 20% response distribution. A total of 106 individuals from the study population of 200 participated, provided they met the eligibility criteria of being ED physicians working in shifts in the Western region of Saudi Arabia.

Data collection

Data were collected using an electronic online-based questionnaire. The questionnaire included the following variables: demographic characteristics, nature of the job, shift characteristics, and the Insomnia Severity Index tool [18]. This tool is a self-report questionnaire consisting of seven items, which evaluates the nature, severity, and impact of insomnia. It was developed by Bastien et al. in 2001 and has been validated in various languages, including Arabic. Each item is rated on a 5-point Likert scale, and the total score ranges from 0 to 28. Higher scores indicate more severe insomnia. The tool has been shown to have excellent internal consistency, test-retest reliability, and convergent validity with other measures of insomnia severity.

Data analysis

Data were analyzed using the statistical package for the social sciences (SPSS, version 29.0). Proportions and frequencies were used to summarize categorical variables, while mean ± SD was used for numerical variables. The Chi-square test was used for the bivariate analysis of categorical outcomes. P-values less than 0.05 were considered significant and a 95% confidence level was used to make inferences.

Ethical considerations

Ethical approval was obtained from the research ethics committee of King Abdullah International Medical Research Center (KAIMRC) in Jeddah city before data collection (NRM22M/014/10). A statement explaining the nature and the purpose of the study was included to gain the participants' consent before filling out the questionnaire. All data were handled anonymously, securely saved, and used for research purposes only.

## Results

A total of 106 physicians participated in the study and were included in the analysis. Table 1 shows the demographic characteristics of emergency room physicians. The majority of the physicians were from Saudi Arabia (98.1%) and worked in government hospitals (94.3%). Most of the physicians were between the ages of 20 and 29 (74.5%), and nearly half of them were male (54.7%). Notably, more than half of the physicians were single (51.9%), while 45.3% were married. The city with the highest number of physicians was Jeddah (81.1%), followed by Taif (7.5%), Makkah (5.7%), and Medina (5.7%). In terms of residency level, most physicians were in their fourth year of residency (36.8%), followed by PGY-2 (23.6%), PGY-3 (22.6%), and PGY-1 (17.0%). Finally, the majority of physicians reported drinking one to two cups of coffee per day (64.2%) and not consuming energy drinks (83.0%).

**Table 1:** Demographic characteristics of ER physicians PGY: Post graduate year

n=106		N	%
Age	20 – 29 y/o	79	74.5
	30 – 39 y/o	26	24.5
	40 – 49 y/o	1	0.9
Gender	Female	48	45.3
	Male	58	54.7
Nationality	Non-Saudi	2	1.9
	Saudi	104	98.1
Marital status	Single	55	51.9
	Married	48	45.3
	Divorced	3	2.8
City	Makkah	6	5.7
	Jeddah	86	81.1
	Medina	6	5.7
	Taif	8	7.5
Residency level	PGY-1	18	17.0
	PGY-2	25	23.6
	PGY-3	24	22.6
	PGY-4	39	36.8
Hospital	Private	6	5.7
	Governmental	100	94.3
Coffee cups	None	19	17.9
	1-2 cups	68	64.2
	≥3	19	17.9
Energy drinks	None	88	83.0
	1-2 cups	16	15.1
	≥3	2	1.9
Smoking status	Yes	35	100.0
Psychiatric diagnosis	Yes	10	100.0

Table 2 provides information on the shift patterns of the ER physicians. Most of the physicians (89.6%) reported having 13-17 shifts per month, and the majority (92.5%) had at least 11 hours of rest time between shifts. About 70% of the physicians had three-to-four night shifts per month.

**Table 2:** Shift patterns of ER physicians

n=106		N	%
Number of working shifts per month	8 – 12	7	6.6
	13 – 17	95	89.6
	18 – 21	4	3.8
Night shifts per month	2 or fewer nights	1	0.9
	3-4	74	69.8
	≥5	31	29.2
Rest time between shifts	Less than 11 hours	8	7.5
	11 hours or more	98	92.5

Table 3 displays the association between insomnia severity and various factors, providing insightful information about the relationship between insomnia and age. The study discovered that insomnia severity had a statistically significant association with age (p=0.025). Specifically, younger physicians reported higher levels of insomnia severity compared to their older colleagues. This finding suggests that age may play a significant role in the development of insomnia, and younger individuals may be more susceptible to the condition.

Despite this statistically significant association between insomnia and age, the study did not find any other significant associations between insomnia severity and other demographic or work-related factors. Specifically, there were no significant associations found between insomnia severity and factors such as gender, nationality, marital status, smoking status, coffee consumption, energy drink consumption, number of shifts per month, night shifts per month, and rest time between shifts.

**Table 3:** Associated factors with insomnia severity Chi-square test, Fisher Exact test

n=106		Insomnia Severity Index					
		No clinically significant insomnia	Subthreshold insomnia	Clinical insomnia moderate severity	Clinical insomnia severe	P-value	
		N (%)	N (%)	N (%)	N (%)		
	Total	19 (17.5)	49 (46.2)	35 (33.0)	3 (2.8)		
Age	20 – 29 y/o	16 (84.2)	41 (83.7)	19 (54.3)	3 (100.0)	0.025*	
	30 – 39 y/o	3 (15.8)	8 (16.3)	15 (42.9)	0 (0.0)		
	40 – 49 y/o	0 (0.0)	0 (0.0)	1 (2.9)	0 (0.0)		
Gender	Female	9 (47.4)	24 (49.0)	13 (37.1)	2 (66.7)	0.631	
	Male	10 (52.6)	25 (51.0)	22 (62.9)	1 (33.3)		
Marital status	Single	11 (57.9)	29 (59.2)	14 (40.0)	1 (33.3)	0.224	
	Married	8 (42.1)	20 (40.8)	18 (51.4)	2 (66.7)		
	Divorced	0 (0.0)	0 (0.0)	3 (8.6)	0 (0.0)		
Smoking status	No	14 (73.7)	33 (67.3)	22 (62.9)	2 (66.7)	0.878	
	Yes	5 (26.3)	16 (32.7)	13 (37.1)	1 (33.3)		
Coffee cups	None	3 (15.8)	9 (18.4)	7 (20.0)	0 (0.0)	0.998	
	1-2 cups	13 (68.4)	30 (61.2)	22 (62.9)	3 (100.0)		
	≥3	3 (15.8)	10 (20.4)	6 (17.1)	0 (0.0)		
Energy drinks	None	16 (84.2)	44 (89.8)	26 (74.3)	2 (66.7)	0.083	
	1-2 cups	3 (15.8)	3 (6.1)	9 (25.7)	1 (33.3)		
	≥3	0 (0.0)	2 (4.1)	0 (0.0)	0 (0.0)		
City	Makkah	2 (10.5)	3 (6.1)	1 (2.9)	0 (0.0)	0.851	
	Jeddah	14 (73.7)	38 (77.6)	31 (88.6)	3 (100.0)		
	Medina	1 (5.3)	3 (6.1)	2 (5.7)	0 (0.0)		
	Taif	2 (10.5)	5 (10.2)	1 (2.9)	0 (0.0)		
Hospital	Private	0 (0.0)	3 (6.1)	3 (8.6)	0 (0.0)	0.636	
	Governmental	19 (100)	46 (93.9)	32 (91.4)	3 (100.0)		
Residency level	PGY-1	7 (36.8)	8 (16.3)	3 (8.6)	0 36.8	0.199	
	PGY-2	3 (15.8)	13 (26.5)	7 (20.0)	2 (66.7)		
	PGY-3	3 (15.8)	12 (24.5)	8 (22.9)	1 (33.3)		
	PGY-4	6 (31.6)	16 (32.7)	17 (48.6)	0 (0.0)		
Number of shifts per month	8 – 12	3 (15.8)	1 (2.0)	3 (8.6)	0 (0.0)	0.102	
	13 – 17	16 (84.2)	46 (93.9)	31 (88.6)	2 (66.7)		
	18 – 21	0 (0.0)	2 (4.1)	1 (2.9)	1 (33.3)		
Night shifts per month	2 or fewer nights	1 (5.3)	0 (0.0)	0 (0.0)	0 (0.0)	.086	
	3 – 4	10 (52.6)	35 (71.4)	28 (80.0)	1 (33.3)		
	≥5	8 (42.1)	14 (28.6)	7 (20.0)	2 (66.7)		
Rest time between shifts	Less than 11 hours	0 (0.0)	4 (8.2)	3 (8.6)	1 (33.3)	0.238	
	11 hours or more	19 (100.0	45 (91.8)	32 (91.4)	2 (66.7)		

## Discussion

Sleep is an integral part of one’s daily life as it comprises at least one-quarter of a person’s full day, and the better the sleep hygiene and quality, the better the achieved quality of life. Shift workers, especially ED physicians, are at higher risk for sleep disturbances such as insomnia compared to the general population due to their changing working hours. In the current study, 106 ED physicians were evaluated for the presence of insomnia. Around 98% of the participating physicians are Saudi, and approximately 95% of them have worked in government hospitals. As for the gender and marital status, it is distributed almost equally.

As per the cross-sectional study that was published by Yih-Farng et al. evaluating the risk of insomnia and hypnotic use among ED physicians, which enrolled 1,097 ED physicians, 14,112 non-ED physicians, and 4,388 general population in the study, they reported the prevalence of insomnia among ED physicians, non-ED physicians, and the general population to be 5.56%, 4.08%, and 1.73% respectively [19]. Moreover, another survey-based study performed in Japan by Chiba et al. that involved 816 ED physicians concluded that the prevalence of chronic insomnia and sleep aid use among ED physicians is approximately 24% [20]. As for the factors that have been found to be associated with insomnia according to the aforementioned study, long working hours and stress were the most prevalent [20]. As per the current study, the prevalence of insomnia among the participating ED physicians was found to be 35.8% ranging from moderate to severe clinical insomnia, and the only factor that was found to have a statistically significant association with insomnia severity is age (p=0.025). In fact, younger physicians experienced severe insomnia compared to their older colleagues, which could suggest that age itself plays a role and/or that older ED physicians have more experience dealing with stress, thus having less severe forms of insomnia. However, the age distribution in the study sample might play a role in these findings since most participants (74.5%) fall in the 20-29 years old age category.

Furthermore, these sleep disturbances can lead to deleterious effects on their lives, since as reported by Brian Ferguson et al. in their study evaluating sleep deficits among ED physicians, around one-third of the participants reported a history of falling asleep on the way back home, and 53% of them reported difficulty falling asleep mostly before night shifts, which is most likely pertained to disturbances in the normal circadian rhythm [21]. According to Kim et al., circadian rhythm disturbance is found to exhibit a negative effect on cortisol by increasing its secretion and decreasing the secretion of melatonin as well as disturbing glucose and lipid homeostasis which in turn will lead to increased sleep latency and thus resulting in poor sleep quality and hygiene [22]. Thus, the prevalence of sleeping aids usage has increased among ED physicians and shift workers, in fact, a cross-sectional study performed among ED physicians in Canada reported that 54% of participants are using sleeping aids in order to cope with their sleeping difficulties [23]. This was further supported by another study performed in Australia which reported that 46.5% of ED doctors are using sleeping aids such as melatonin, benzodiazepine, and pseudoephedrine to manage their sleep and improve their overall performance [24].

This study's findings suggest that interventions should be created to address the high prevalence of insomnia among ED physicians in Saudi Arabia. Such interventions may include education and training on sleep hygiene and stress management, regular screening for insomnia, and access to appropriate treatment options, such as cognitive-behavioral therapy for insomnia (CBT-I) or medication when necessary. Furthermore, since age may play a role in the development of insomnia, it may be advantageous to implement targeted interventions for younger physicians to address this issue.

This study has a few limitations that should be considered when interpreting the findings. Firstly, the study used a cross-sectional design, which means that we cannot establish causality or temporal relationships between variables. Secondly, the study sample was limited to ED physicians in the Western region of Saudi Arabia, so it may not be representative of all physicians in the country or other regions. Furthermore, the age distribution in the sample was disproportional between age categories. Finally, the study relied on self-reported data, which may introduce bias or inaccuracies in the measurement of insomnia severity.

## Conclusions

In conclusion, this study aimed to assess insomnia and its associated factors among ED physicians in Saudi Arabia. The study found a high prevalence of insomnia among ED physicians, with 35.8% of participants having moderate to severe clinical insomnia. Additionally, younger physicians experienced more severe insomnia than their older colleagues, highlighting the potential role of age in the development of insomnia. Although no significant associations were found between insomnia severity and other demographic or work-related factors, these findings emphasize the need for further research and interventions to address the high prevalence of insomnia among ED physicians. Improving the sleep quality of ED physicians is crucial not only for their health and well-being but also for the safety and quality of care provided to their patients.
